# Advancing optothermal manipulation: decoupling temperature and flow fields in quasi-isothermal microscale streaming

**DOI:** 10.1038/s41377-023-01246-8

**Published:** 2023-08-31

**Authors:** Youngsun Kim, Yuebing Zheng

**Affiliations:** 1 Materials Science and Engineering Program and Texas Materials Institute, The University of Texas at Austin, Austin, TX 78712 USA; 2 Walker Department of Mechanical Engineering, The University of Texas at Austin, Austin, TX 78712 USA

**Keywords:** Optical manipulation and tweezers, Optofluidics

## Abstract

By decoupling temperature and flow fields through symmetry-correlated laser scan sequences, ISO-FLUCS enables quasi-isothermal optofluidic microscale streaming. This technique offers precise control over fluid manipulation while minimizing thermal damage.

## Abstract (web summary)

Quasi-isothermal optofluidic streaming is enabled by symmetry-correlated laser scan sequences.

## Graphical abstract

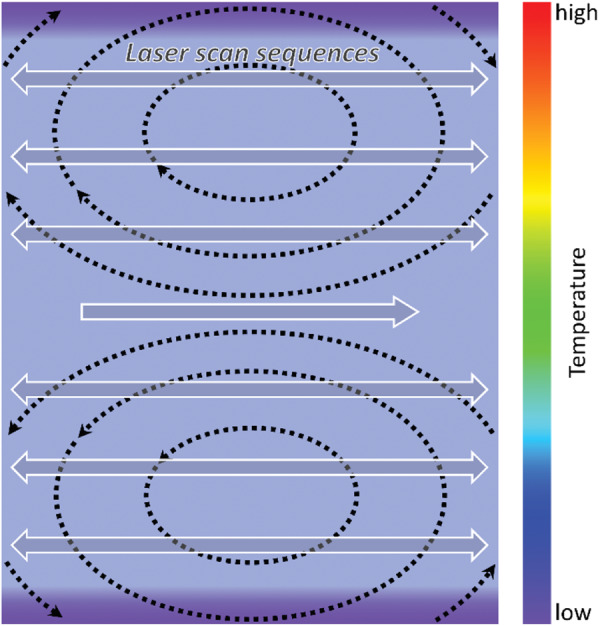

Manipulating matter on a micro/nanoscale has garnered significant interest across various fields^1–3^. Optothermal systems, which utilize optical stimuli to generate heat and induce motions of matter, have proven effective for micro/nanomanipulation^4,5^. Compared to purely optical manipulation, optothermal techniques provide expanded working modes and the ability to target a diverse range of materials at reduced optical power requirements.

One of the established optothermal techniques is focused-light-induced cytoplasmic streaming (FLUCS), which was developed to manipulate cytoplasmic flows within cells through the optical control of thermoviscous flows^6^. FLUCS involves using a moving mid-infrared laser to locally heat the cytoplasm. The heating induces cytoplasmic flows through thermoviscous expansion phenomena, which can be modulated by laser scanning paths. FLUCS has provided valuable insights into cellular properties and processes, such as cytoplasmic microrheology, cell division, and cell death, by offering a means to perturb cytoplasmic flows^6–8^.

However, when heat-mediated micromanipulation techniques are applied to biological specimens, the potential for thermal damage becomes a major concern. Many cell components and cells themselves are heat-sensitive in terms of their physiological and physicochemical properties^9–11^. In living organisms, where complex interactions occur, variations in absolute temperature and temperature gradients may result in undesired effects that deviate from normal physiological conditions.

Addressing this concern, Moritz Kreysing and co-workers developed a micromanipulation system that mitigates thermal damage^12^. Their innovation was focused on the scanning paths used in FLUCS. By introducing additional back-and-forth scan paths near the original flow-inducing path, they could flatten the time-average temperature gradient (Fig. 1). The authors extensively studied how the spatiotemporal symmetry relations of scan sequences influenced the resultant temperature and flow profiles, ultimately deriving optimal scan sequences for achieving quasi-homogenous temperature distribution while establishing desired flow patterns. This approach is referred to as isothermal FLUCS (ISO-FLUCS). With this scan-sequence-based approach, they successfully demonstrated intracellular streaming in *C. elegans* with temperature homogeneity.

**Fig. 1:**
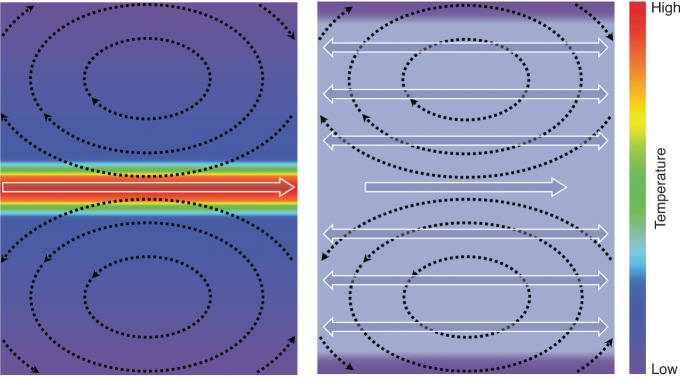
Schematic illustrations of focused-light-induced streaming and quasi-isothermal focused-light-induced streaming. Left: focused-light-induced streaming with a single laser scan. Right: quasi-isothermal focused-light-induced streaming with additional sequential laser scans. Solid arrows with white outlines and dotted back arrows indicate laser scan paths and induced thermoviscous flows, respectively

The key advantage of ISO-FLUCS is the decoupling of the temperature field from the flow field. In most optothermal manipulation scenarios, these two fields are strongly coupled, as the temperature gradient serves as a major driving force for the manipulation. However, by designing symmetry-correlated scan sequences that eliminate temperature gradients and non-principal flow components, it becomes possible to effectively disentangle the temperature and flow fields. This decoupling suggests that similar additive-destructive strategies could be considered in other optothermal manipulation systems that rely on a single pair of stimulus and response, especially when targeting thermally delicate specimens. From a fundamental standpoint, patterned and complex optical stimuli could be adapted to diversify the operation modes of optothermal manipulation.

Further implementation of ISO-FLUCS in vitro and in vivo may require additional improvements and developments. Cells and living organisms possess complicated cytoplasmic environments that exhibit intrinsic heterogeneity both within the same cell type and across different cell types. Given that scan sequences in ISO-FLUCS are influenced by material properties such as the temperature dependence of viscosity and density, variations in these properties need to be considered when determining parameters for laser scan sequences. Particularly, if the physical properties of a given sample can be measured in situ and feedback control is integrated to adjust scan sequences accordingly, a more comprehensive and reliable cytoplasmic streaming system can be realized.

In summary, ISO-FLUCS represents a significant breakthrough in the field of optothermal manipulation. By decoupling the temperature and flow fields, this technique opens new possibilities for controlled fluid manipulation. As researchers continue to refine and expand upon this technique, its exciting applications in various fields, from biotechnology to analytical chemistry, are expected in the years to come.
